# Structural empowerment of nurses in the hospital setting*

**DOI:** 10.1590/1518-8345.3915.3373

**Published:** 2020-11-06

**Authors:** Lenize Nunes Moura, Silviamar Camponogara, José Luís Guedes Dos Santos, Renata Cristina Gasparino, Rosângela Marion Da Silva, Etiane De Oliveira Freitas

**Affiliations:** 1 Universidade Federal de Santa Maria, Santa Maria, RS, Brazil.; 2 Scholarship holder at the Coordenação de Aperfeiçoamento de Pessoal de Nível Superior (CAPES), Brazil.; 3 Universidade Federal de Santa Catarina, Florianópolis, SC, Brazil.; 4 Universidade Estadual de Campinas, Faculdade de Enfermagem, Campinas, SP, Brazil.

**Keywords:** Health Facility Environment, Hospital Administration, Nursing, Efficiency, Organization and Administration, Power, Ambiente de Instituições de Saúde, Administração Hospitalar, Enfermagem, Eficiência, Organização e Administração, Poder, Ambiente de Instituciones de Salud, Administración Hospitalaria, Enfermería, Eficiencia, Organización y Administración, Poder

## Abstract

**Objective:**

to measure the level of structural empowerment of nurses working in a university hospital.

**Method:**

a descriptive, analytical, and cross-sectional study, carried out with 237 nurses, who developed care and management activities. Data collection took place through a self-administered questionnaire with questions on the personal and professional characterization and the Work Effectiveness Conditions Questionnaire II. Data analysis used descriptive and inferential statistics.

**Results:**

it was identified that nurses have a moderate level of structural empowerment (18.06±SD 0.9). The greatest value was obtained in the Opportunity dimension (4.08*±*SD 0.8), followed by the Resources (3.17*±*SD 0.8) and Informal power (3,04*±*SD 0.9) dimensions; while the scores of Support (2.67*±*SD 1.0), Formal power (2.59±SD 0.9), and Information (2.51*±*SD 0.9) were lower.

**Conclusion:**

the level of structural empowerment of the nurses was moderate, which means partial access to opportunities, resources, support, and information of the institution.

## Abstract (pt)

**Objetivo:**

mensurar o nível de empoderamento estrutural dos enfermeiros de um hospital universitário.

**Método:**

estudo descritivo analítico, transversal, realizado com 237 enfermeiros, que desenvolviam atividades assistenciais e gerenciais. A coleta de dados ocorreu por meio de um questionário autoaplicável com questões de caracterização pessoal e profissional e Questionário de Condições de Eficácia no Trabalho II. A análise dos dados utilizou-se da estatística descritiva e inferencial.

**Resultados:**

identificou-se que os enfermeiros possuem um nível moderado de empoderamento estrutural (18,06±dp 0,9). O maior valor foi obtido na dimensão Oportunidade (4,08*±*dp 0,8), seguido pelas dimensões Recursos (3,17*±*dp 0,8) e Poder informal (3,04*±*dp 0,9); enquanto as pontuações de Suporte (2,67*±*dp1,0), Poder formal (2,59±dp 0,9) e Informações (2,51*±*dp 0,9) foram as mais baixas.

**Conclusão:**

o nível de empoderamento estrutural dos enfermeiros foi moderado, o que significa acesso parcial às oportunidades, aos recursos, ao suporte e as informações da instituição.

## Abstract (es)

**Objetivo:**

medir el nivel de empoderamiento estructural de los enfermeros de un hospital universitario.

**Método:**

estudio descriptivo, analítico, transversal, realizado con 237 enfermeros, que desarrollaron actividades de atención y gestión. La recopilación de datos se realizó mediante un cuestionario autoadministrado con consignas de caracterización personal y profesional e Cuestionario de Condiciones de Efectividad en el Trabajo II. En el análisis de datos se utilizó la estadística descriptiva e inferencial.

**Resultados:**

se identificó que los enfermeros poseen un nivel moderado de empoderamiento estructural (18,06±de 0,9). El valor más alto se obtuvo en la dimensión Oportunidad (4,08*±*de 0,8), seguido de las dimensiones Recursos (3,17*±* de 0,8) y Poder informal (3,04 *±*de 0,9); mientras que los puntajes en Soporte (2,67 *±*dp 1,0), Poder formal (2,59±de 0,9) e Información (2,51*±*de 0,9) fueron los más bajos.

**Conclusión:**

el nivel de empoderamiento estructural de los enfermeros fue moderado, lo que significa acceso parcial a las oportunidades, los recursos, el apoyo y las informaciones de la institución.

## Introduction

The hospital work environment is constantly changing to keep up with technological advances and to develop strategies aimed at providing quality and safe care to the patients^(^ 1 ^)^. This scenario requires health workers to be proactive and to constantly seek professional updating and development, especially by nurses^(^ 1 ^-^ 2 ^)^.

In the context of the organization of hospital work, nurses are responsible for the management of care and of the nursing services, which involves the development of leadership practices, conflict management, staff sizing, provision and forecasting resources^(^ 2 ^)^. For the proper performance of these actions, it is important that the organizations adopt models and management policies that enhance the empowerment of nurses^(^ 3 ^-^ 4 ^)^.

In general, empowerment is the process by which individuals strengthen or develop skills to promote positive changes in the context in which they are inserted. In the nursing literature, empowerment is used as a comprehensive concept to describe elements of professional growth and development^(^ 4 ^)^. In the organizational context, there are two different theoretical conceptions of empowerment: (1) Structural empowerment and (2) Psychological empowerment^(^ 4 ^-^ 5 ^)^.

Structural empowerment refers to the ability to mobilize resources and achieve goals through access to information, support, resources, and opportunities. Access to information refers to the knowledge of organizational changes and policies, as well as to the technical knowledge necessary to carry out the work. Nurses gain access to support by receiving feedback and guidance from subordinates, colleagues, and superiors, which enables autonomous decision making. Access to resources involves the nurses’ ability to obtain the supplies, resources, and materials needed to achieve the organizational goals. The opportunities refer to the possibilities of learning and professional development of nurses^(^ 5 ^-^ 6 ^)^.

Psychological empowerment is the worker’s psychological response to empowerment arising from working conditions, which generates motivation and contributes to the sense of personal control in the workplace. In Nursing, psychological empowerment involves the perception of the professional about the valorization of their work and their contributions to contribute to the care processes, which leads to a feeling of competence and freedom. Thus, structural empowerment leads to nurses’ psychological empowerment^(^ 6 ^-^ 7 ^)^.

Thus, the empowerment of nurses contributes to greater professional satisfaction, decreased burnout rates, and increased autonomy and organizational commitment. In addition, it positively impacts patient safety and quality of care in the health services^(^ 3 ^-^ 4 ^,^ 6 ^-^ 8 ^)^. As a result, it is necessary for the managers to be attentive to their organizational practices, since just ensuring the quality of the technical aspects of processes or services is no longer sufficient to maintain the efficiency of organizations, and it is also necessary to invest in the involved people^(^ 9 ^)^.

A number of studies on the structural empowerment of nurses have been developed in several countries in the last decade, mainly the United States of America, Canada, China, United Kingdom, Germany, Italy, Spain, and Portugal^(^ 4 ^,^ 10 ^-^ 11 ^)^. In Brazil, this theme is still incipient, despite the recent growth of research on nurses’ working environment^(^ 12 ^)^.

It is believed that one of the reasons for the lack of studies on nurses’ empowerment is the number of instruments available to measure this variable in the national scenario. The Conditions of Work Effectiveness-Questionnaire-II (CWEQ-II), the main research instrument used internationally, for example, was validated for the population of Brazilian nurses in 2013^(^ 13 ^)^. However, in database searches, studies on the structural empowerment of nurses in Brazil were not identified.

Thus, in view of the importance of nurses’ empowerment in contemporary health organizations and the need to advance studies on the theme in Brazil, this study was designed with the following research question: What are the levels of structural empowerment of nurses in a university hospital? The measurement of empowerment levels can support the development of strategies to improve the nurses’ work environment, resulting in greater professional satisfaction and, consequently, in improving the quality of care for the treated patients.

The aim of this study was to measure the level of structural empowerment of nurses working in a university hospital.

## Method

A descriptive, analytical, and cross-sectional study carried out in a large public university hospital with 403 beds, located in southern Brazil. As a teaching hospital, the institution focuses on the development of teaching, research and health care, serving 100% of the patients through the Unified Health System (*Sistema* Único *de Saúde*, SUS).

The study participants were nurses who performed care and management activities in the different sectors of the institution: ambulatory, emergency, diagnostic support, intensive care, inpatient unit, surgery, and sterilization. The following inclusion criteria were considered: being in the professional practice at the time of data collection and working in the selected unit for at least three months. The demarcation of this period was based on the assumption that this time would be the minimum for the nurse to be adapted to the work sector and, thus, could contribute more effectively to the research. As exclusion criteria, the following were considered: being on vacation, leave or absence for any reason during the data collection period, and/or having a direct link with the research group promoting the investigation.

For defining the research participants, a convenience sample was used. However, the minimum sample criterion was adopted to avoid bias in selecting the study subjects. To estimate the sample size, a 95% confidence level and a 5% margin of error were used. Thus, when applying the minimum sample calculation, of the total of 336 nurses at the institution, a minimum of 180 participants was obtained to compose the sample.

297 nurses were invited to participate in the study. Each of them was approached individually at their workplace. However, 22 nurses did not meet the inclusion and exclusion criteria. Another 38 subjects refused to participate in the research or did not return the questionnaire. Thus, the final sample of the study was composed of 237 nurses, which corresponds to 79.79% of the population initially accessed and includes the minimum number of participants, according to the aforementioned sample calculation.

Data was collected from February to June 2018, using a self-administered questionnaire, which was printed out for each participant during their work shift. The questionnaire contained two parts. The first was composed by questions of sociodemographic characterization (age, gender, and marital status) and professional characterization (year of graduation, professional training, length of experience in the profession, work shift, working time at the unit and at the institution, weekly workload, employment relationship, and existence of another employment relationship). In the sequence, there was the adapted and validated version for use in Brazil of the Conditions of Work Effectiveness Questionnaire-II (CWEQ-II)^(^ 10 ^)^.

The CET-II is an instrument of public domain, which aims to measure components of structural empowerment found in the nurse’s work environment^(^ 13 ^)^. It consists of 21 items, divided into six components: (1) Opportunity - feeling of challenge and opportunity to learn and grow, (2) Information - data, knowledge and experience, awareness of the organization›s objectives (mission), (3) Support - feedback and guidance received from superiors, peers and subordinates, (4) Resources - time, materials and equipment to achieve organizational goals, (5) Formal power - jobs that provide flexibility and visibility and that are relevant to the main organizational processes and (6) Informal power - network of alliances with colleagues and subordinates, inside and outside the organization^(^ 4 ^)^. It also has a construct of global empowerment, containing two items that validate it, which were not used in this study.

The measurement scale of the CET-II is of the Likert type, with scores varying from 1 (nothing) to 5 (much). The items for each component are added together and averaged to provide a score ranging from 1 to 5 for each component. The sum of the components can vary from 6 to 30, with values between 6 and 13 meaning low levels of empowerment, between 14 and 22 meaning moderate levels of empowerment and values between 23 and 30 representing high levels of empowerment, that is, high scores represent greater perceptions of empowerment^(^ 14 ^)^.

The data were organized by double independent typing in an electronic spreadsheet in the form of a database, using the Excel - Windows/XP program and analyzed using the Statistical Package for the Social Sciences (SPSS), version 21. To describe the profile of the sample and of empowerment, descriptive statistics were used with the elaboration of tables of absolute frequency (n) and percentage (%) of the categorical variables and measures of central tendency and dispersion for continuous variables.

The internal consistency of the scale was assessed by the Cronbach’s alpha coefficient, which was calculated for each of the subscales and for the total items of the instrument. In this study, the alpha value was 0.7 for the scales of opportunity, resources, and formal and informal power. The information and support scale was 0.8 and the total number of items in the CET-II was 0.9. It is noteworthy that the Cronbach’s alpha value can vary from zero to one. Thus, the higher the value, the greater the internal consistency of the instrument^(^ 15 ^)^.

Data distribution normality was assessed using the Kolmogorov-Smirnov test. For comparing the CET-II among the hospital sectors, the Kruskall-Wallis test was performed. When statistical differences were evidenced, the Dunn Method was applied to determine which groups showed such differences. The level of significance was set at 5% (*p*<0.05).

The research is linked to a multicenter macro-project about the work environment and empowerment of nurses in the hospital context. It was approved by the Research Ethics Committee, under opinion number 2,465,337, and complied with the provisions of Resolution No. 466/2012 of the National Health Council^(^ 16 ^)^.

## Results

Of the 237 nurses, 209 were female (88.6%), with a mean age of 38 years old, 187 (78.9%) had a partner, 139 (58.6%) had a Specialist title, and 140 (59.1%) had their employment contract governed by the Consolidation of Labor Laws (*Consolidação das Leis do Trabalho*, CLT).

The mean time of experience in the profession was 13.0 years (SD±8.0), the mean time of work in the institution was 8 years (SD±7.9) and, in the sector, it was 4.9 years (SD±5.3). Most of these professionals, 215 (90.7%), reported having only one job and had a mean weekly workload of 36 hours (Table 1).

**Table 1:** Socio-professional characterization of the study participants (237). Santa Maria, RS, Brazil, 2018

Variables	N	%	Mean ± SD*
Age (years old)			38 ± 8.57
Gender (n=236)			
Female	209	88.6	
Male	27	11.4	
Marital status (n=234)			
Has a partner	187	78.9	
No partner	47	19.8	
Training (n=237)			
Graduation	24	10.1	
Specialization	139	58.6	
Master’s degree	62	26.2	
PhD	9	3.8	
Did not answer	3	1.3	
Employment contract (n=234)			
CLT	140	59.1	
RJU	94	39.7	
Other employment contract (n=237)			
No	215	90.7	
Yes	22	9.3	
Work shift (n=237)			
Night	72	30.4	
Afternoon	59	24.9	
Morning	57	24.1	
Mixed (morning, afternoon, and night)	49	20.7	
Professional experience (years)	237		13 ± 8.0
Experience in the hospital (years)	236		8 ± 7.9
Experience in the sector (years)	236		4.9 ± 5.3
Number of patients per work shift	212		19.9 ± 16.9
Weekly workload (hours)	224		36 ± 7.6

* SD = Standard deviation

The total mean of the CET-II, which corresponds to the sum of the means of each component of structural empowerment, was (18.06±0.9), resulting in a moderate level of structural empowerment of the nurses participating in the study. The complete results are described in Table 2.

**Table 2:** Means and standard deviation of each component and sum of the means of the CET-II. Santa Maria, RS, Brazil, 2018

Domains/Items	Mean	SD*
**Opportunity**	**4.08**	**0.8**
Work is challenging	4.05	1.0
Opportunity to gain new skills and knowledge	3.95	0.9
Tasks that require all my skills and knowledge	4.23	0.9
**Information**	**2.51**	**0.9**
About the current condition of the hospital	2.81	1.0
About the values of the hospital administration	2.35	1.1
The objectives of hospital administration	2.37	1.1
**Support**	**2.67**	**1.0**
Specific comments on what you do well	2.54	1.2
Specific comments on what you could improve	2.60	1.1
Useful tips or troubleshooting advice	2.87	1.1
**Resources**	**3.17**	**0.8**
Time available to carry out bureaucratic work	3.04	0.9
Time available to fulfill job requirements	3.40	0.8
Obtaining temporary assistance when needed	3.07	1.1
**Formal Power**	**2.59**	**0.9**
The rewards for innovation at work are:	2.13	1.1
The flexibility in my work is:	3.09	1.0
The visibility of my work activities within the institution is:	2.53	1.1
**Informal Power**	**3.04**	**0.9**
Participating with physicians in the patient care	3.13	1.1
Being sought after by your peers to help solve problems	3.68	1.0
Being approached by administrators to help with problems	2.28	1.2
Seeking out ideas from professionals other than physicians, e.g., Physiotherapists, Occupational Therapists, Dieticians.	3,07	1,2
**TOTAL**	**18.06**	**0.9**

* SD = Standard deviation

Structural empowerment was assessed according to the hospital sectors by means of means, maximum and minimum, and standard deviation (Table 3). Dunn’s test identified a significant difference in the opportunity domain between the emergency room and intensive care unit (ICU) sectors. The other sectors showed no significant difference.

**Table 3:** Conditions of Efficiency at Work CET-II according to the hospital sectors. Santa Maria, RS, Brazil, 2018

Domains/Sectors		Mean	Minimum	Maximum	SD*
**Opportunity** p=0.0025^†^	Ambulatory	4.14	3.67	5.00	0.5
	Management Activities	4.33	3.00	5.00	0.7
	Surgical and Obstetric	4.15	2.33	5.00	0.7
	Hematology-Oncology	4.12	2.00	5.00	0.9
	**Emergency Room**	**4.54**	2.67	5.00	0.6
	Psychiatry	4.46	4.00	5.00	0.3
	Support Services	3.72	2.00	4.67	0.9
	Hospitalization Units	4.03	1.67	5.00	0.9
	**Intensive Care Units**	**3.85**	2.33	5.00	0.8
**Information** p=0.3244^†^	Ambulatory	2.04	1.00	4.00	0.9
	Management Activities	2.89	1.00	4.33	1.1
	Surgical and Obstetric	2.71	1.00	5.00	0.9
	Hematology-Oncology	2.65	1.00	4.33	0.9
	Emergency Room	2.54	1.00	5.00	1.0
	Psychiatry	3.33	1.33	5.00	1.1
	Support Services	2.39	1.00	3.33	0.6
	Hospitalization Units	2.49	1.00	4.67	0.9
	Intensive Care Units	2.22	1.00	5.00	0.9
**Support** p=0.2082^†^	Ambulatory	2.46	2.00	3.33	0.5
	Management Activities	3.24	1.67	5.00	1.0
	Surgical and Obstetric	2.62	1.00	4.33	1.0
	Hematology-Oncology	3.13	1.33	5.00	0.9
	Emergency Room	2.58	1.00	5.00	1.1
	Psychiatry	3.50	2.00	4.00	0.7
	Support Services	2.50	1.33	4.33	0.8
	Hospitalization Units	2.46	1.00	4.67	0.9
	Intensive Care Units	2.46	1.00	5.00	0.9
**Resources** p=0.0743^†^	Ambulatory	2.75	1.67	3.33	0.5
	Management Activities	3.60	2.00	5.00	0.8
	Surgical and Obstetric	3.00	2.00	5.00	0.6
	Hematology-Oncology	3.64	2.00	4.67	0.6
	Emergency Room	2.82	1.00	4.67	0.8
	Psychiatry	3.54	2.33	5.00	0.8
	Support Services	3.06	1.67	4.67	0.8
	Hospitalization Units	2.95	1.67	4.67	0.7
	Intensive Care Units	3.34	1.33	5.00	0.8
**Formal Power** p=0.090^†^	Ambulatory	3.17	4.00	2.67	0.4
	Management Activities	2.93	5.00	1.00	1.1
	Surgical and Obstetric	2.48	4.67	1.00	1.0
	Hematology-Oncology	2.94	4.33	1.00	0.8
	Emergency Room	2.56	4.67	1.00	1.0
	Psychiatry	3.21	4.00	2.33	0.6
	Support Services	2.61	4.00	1.33	0.8
	Hospitalization Units	2.40	4.33	1.00	0.8
	Intensive Care Units	2.36	5.00	1.00	0.9
**Informal Power** p=0.2327^†^	Ambulatory	3.19	3.67	2.00	0.5
	Management Activities	3.36	5.00	1.33	1.1
	Surgical and Obstetric	3.24	5.00	1.00	0.9
	Hematology-Oncology	3.15	5.00	1.33	0.8
	Emergency Room	3.12	4.67	1.33	0.9
	Psychiatry	3.75	5.00	3.00	0.7
	Support Services	3.14	4.67	1.67	1.0
	Hospitalization Units	2.85	4.67	1.00	0.9
	Intensive Care Units	2.82	5.00	1.33	0.9

* SD = Standard deviation;

† Kruskall-Wallis Test (5% significance level :*p*<0.05).

From the sum of the means, it was verified that the level of structural empowerment in all hospital sectors was moderate, with the Psychiatry sector having the highest sum of the means and the Intensive Care Units, the lowest (Table 4).

**Table 4:** Sum of the means and levels of structural empowerment, according to the hospital sectors. Santa Maria, RS, Brazil, 2018

Sectors	Sum of the means	Levels of empowerment
Ambulatory	17.7	Moderate Level
Management Activities	20.3	Moderate Level
Surgical and Obstetric	18.2	Moderate Level
Hematology-Oncology	19.6	Moderate Level
Emergency Room	18.1	Moderate Level
Psychiatry	21.7	Moderate Level
Support Services	17.4	Moderate Level
Hospitalization Units	17.1	Moderate Level
Intensive Care Units	17.0	Moderate Level

## Discussion

In this study, the sample of nurses was predominantly female, with a partner. These are similar results to other national^(^ 12 ^-^ 13 ^)^ and international^(^ 17 ^-^ 18 ^)^ surveys.

In relation to vocational training, most of the nurses had graduate degrees being *lato sensu* or *stricto sensu,* which corroborates earlier research outcomes^(^ 18 ^-^ 19 ^)^. The professional has been increasingly seeking professional qualification in order to expand knowledge and meet the demands of the labor market. The continuous search for improvement and updating is essential for the professionals in view of the complexity of procedures and increasingly innovative technologies in the hospital context^(^ 20 ^)^. Regarding empowerment, this data is extremely relevant, because empowering implies having knowledge, attitudes, and ability to efficiently perform their role.

In this study, the participants have a mean experience of 13 years as nurses. On average, the professionals have worked at the institution for eight years and their mean experience in the sector was 4.9 years. These results were similar to those of other studies found in the international literature^(^ 18 ^-^ 19 ^)^.

No studies were found that specifically linked empowerment to professional practice time. However, it is believed that the greater the experience, the greater the empowerment, which allows reading this result in a positive way. Newly graduated nurses often face challenging and stressful situations at work, as they are not always able to successfully complete their activities or receive support to perform certain care actions. This happens not only due to lack of skill, but also due to fear and common insecurity at the beginning of professional life^(^ 21 ^)^.

As for the total mean of the CET-II, the nurses showed a moderate level of structural empowerment. Regarding the domains of empowerment, access to opportunity was the component with the highest mean, followed by the resource and informal power components. The information domain had the worst mean, followed by formal power and by support. These results are similar to a study carried out in Portugal in which the “opportunity” and “informal power” domains had better evaluations and the “formal power” and “information” domains also had worse evaluations^(^ 22 ^)^.

In this study, the results showed that nurses have opportunities to learn and grow professionally, within the work environment. Similar results are described in previous studies using the same instrument, reinforcing that the opportunity to learn and grow at work implies the use of personal skills, promoting a sense of self-efficacy and autonomy^(^ 17 ^-^ 19 ^)^.

When comparing the nine sectors by domain, a significant difference was identified in the opportunity domain, between the emergency room and ICU sectors. That is, the nurses in the emergency room have more opportunity than those in the ICU, in the view of the professionals. The other sectors showed no significant difference; this indicates that, among the other sectors, there is a similarity between access to other resources within the hospital setting.

This result may have been influenced by the characteristic profile of each of the sectors. The ICUs have an intense activity, they are dynamic environments, constantly changing, that attend complex patients and those at risk, demanding a lot of responsibility and commitment from the professionals. In addition, in these units, assistance must be provided through teamwork, in which multiple knowledge and skills need to come together to plan, organize, and define strategies that bring better results to the patients^(^ 23 ^)^.

The emergency room of the researched institution is a reference in urgent and emergency care for a population of more than one million inhabitants. It is the gateway to the hospital service and a place where people seek quick resolutions to health-related problems, when they cannot get resources in primary health care^(^ 24 ^-^ 25 ^)^. It is also noteworthy that the high complexity and unpredictability of the services in this location require the professionals to have a high level of training and the nurse is an important figure, as coordinator of the care team^(^ 26 ^)^.

It is an instigating environment, as it is not limited to a patient and severity profile, making the professional always up to date and seeking knowledge, due to the various types of pathologies that patients have^(^ 25 ^-^ 26 ^)^. Thus, these characteristics make nurses look for more opportunities for growth, learning, and knowledge in this sector.

In the hospital setting, nurses have moderate resources, which corresponds to the time to carry out the bureaucratic work, materials, and equipment necessary to achieve the organizational goals and to fulfill the work requirements^(^ 1 ^,^ 12 ^)^. The sector that obtained the best score in this domain was hematology-oncology. The institution where the research was conducted is a national reference in the pediatric oncology service. It also performs bone marrow transplantation and cancer treatment in general, being a reference in the care of the specialty and qualified as a High Complexity Unit in Oncology in the Services of Radiotherapy, Hematology, and Pediatric Oncology.

It is a specific area of activity, where there is a restricted team and the nursing work is characterized by assistance that involves, in addition to care practices, characteristic situations, such as the specificity of the treatments used and the presence of sensitized patients, with biggest limitations^(^ 10 ^)^. As such, this result can be attributed to several factors, as the institution is a reference in hematology-oncology care, there is a greater possibility that it will follow several safety standards in care, better structure, more resources and equipment, which can offer better conditions for professionals.

An international study conducted with nurses working in intensive care environments revealed that access to the resources was the domain with the lowest score, mainly due to financial restrictions^(^ 17 ^)^. Resource limitations challenge hospitals to improve their quality of performance and to increase the safety and effectiveness of patient care, without providing the best conditions for this^(^ 4 ^)^.

Based on the findings of this study and on the related literature, the hypothesis is raised that nurses with resources and equipment available to provide assistance and management, as well as knowledge of the whole, tend to be more empowered at work, especially psychological empowerment. In addition, in relation to support, the results showed that nurses have moderate support from colleagues and supervisors within the work environment, as well as the resolution of problems and improvements related to work. In this environment, nurses receive feedback on the work performed, as well as guidance from their colleagues, subordinates, and superiors. The more feedback and guidance that the professionals receive from leaders and superiors, the greater the job satisfaction will be^(^ 27 ^)^.

As for formal and informal power, even though both had an intermediate assessment, the latter presented better means in assessing nurses. It is emphasized that formal power refers to rewards and flexibility at work and visibility in activities carried out within the institution. Informal power, on the other hand, deals with the relationships established within the work environment; the results showed that nurses are able to establish a network of alliances with the team of professionals and with the management, making it possible to have a strengthening and visibility within the context working^(^ 13 ^-^ 14 ^)^.

It is worth mentioning that, within an organization, power results from formal precepts (activities that allow for the achievement of organizational objectives) and informal precepts (derived from interpersonal relationships) that allow access to structures that promote empowerment^(^ 18 ^)^. Job satisfaction tends to be higher when you have high levels of empowerment, as well as when you have higher levels of formal and informal power^(^ 28 ^)^.

Although the information domain was moderate, it obtained the lowest value in the investigated location. The study revealed that nurses have moderate information regarding the current conditions in the work environment. A priori, there are two hypotheses for this result. First, the transfer of information from the management is not reaching the professionals, that is, the information is not being passed on; or the nurses are not giving due attention and importance to the information being passed on.

It is essential to check where the failure occurs to correct it. Based on a survey conducted with nurses at a university hospital in Belgium, lack of information became a key challenge for empowerment. The participants pointed out that the information was available, but that it was only aimed at a few colleagues and committees. They also reported the need for better communication, with lack of communication being associated with a failure in management^(^ 29 ^)^.

Another important data pointed out in the present research is related to the fact that the psychiatric sector obtained the best score in terms of access to information, support, formal and informal power. This place demands agility and attention during the service from the professionals, as well as the accomplishment of team work, planning and organization of the activities, and also a specialized technical preparation for better performance in the care of patients^(^ 30 ^)^.

This result may be linked to the particularities of this sector, as it has a work dynamics, where patients are cyclical, hospitalized, and readmitted numerous times due to relapses during treatment, which can result in greater knowledge and mastery, by the professionals, on their working environment.

Thus, it is believed that professionals with full domain, knowledge, and connection with patients have greater safety at work, resulting in greater empowerment. The results also pointed out that the nurses in this sector can have moderately flexibility and visibility in the activities performed within the work environment and have relevant information related to the values and objectives of the hospital administration.

An international study also highlights the importance of nurses’ empowerment for a positive patient safety culture^(^ 31 ^)^. Therefore, it should be noted that managers and nursing leaders must ensure the empowerment of nurses in order to increase the quality of patient care in the hospitals^(^ 28 ^)^.

After the validation of the CET-II for use in Brazil, this is the first study to apply it among nurses at a university hospital. Thus, the data obtained are important for identifying the levels of empowerment of nurses in the hospital environment and discussion about strategies that can contribute to the organization of work in health and nursing aiming at greater satisfaction of professionals and, consequently, better care practices for the patients.

This study provides elements for a better understanding of the structural empowerment of nurses within the work environment. The submitted results may come to contribute to the planning of future interventions in different health care scenarios, especially in the hospital settings. It is recommended that managers and administrators of organizational environments invest in favorable environments to improve the structural empowerment of nurses and, consequently, professional satisfaction and the quality of care provided to the patient. It is suggested that further research be carried out with the intention of expanding the study of the theme and deepening the results.

The main limitations of this study are related to the theme, since the theme of empowerment is still little explored in the Brazilian reality, which hindered a wider discussion of the data with national studies. The research was also carried out in only one public institution, making it necessary to carry out studies in other institutions, enabling the comparison of results and advancing the understanding of the problem at issue.

## Conclusion

From this study, it is concluded that nurses have a moderate perception of structural empowerment in the hospital setting. The Opportunity domain was the component of structural empowerment that had the highest mean score. When comparing hospital sectors, the emergency room showed a higher score in the Opportunity dimension. The psychiatry sector stood out with the best mean values regarding access to information, support, formal and informal power. The hematology-oncology sector has more access to resources.

Thus, this study presents an overview of the structural empowerment of nurses in the hospital setting, being important to conduct new research studies in order to explore the relationship between empowerment and other variables related to the nursing practice. Empowerment is a central element for the nurses’ work, as it predisposes to professional satisfaction and contributes to improving the care provided to the patients.
